# Unmet Social Needs and Breast Cancer Screening Utilization and Stage at Presentation

**DOI:** 10.1001/jamanetworkopen.2023.55301

**Published:** 2024-02-14

**Authors:** Neha Goel, Maya Lubarsky, Alexandra E. Hernandez, Kelley Benck, Emma Lee, Susan Kesmodel, Felicia Knaul, Erin Kobetz, Benjamin O. Anderson

**Affiliations:** 1 Department of Surgery, Division of Surgical Oncology, University of Miami, Miami, Florida; 2 University of Miami Sylvester Comprehensive Cancer Center, Miami, Florida; 3 Harvard T.H. Chan School of Public Health, Harvard University, Boston, Massachusetts; 4 University of Miami Miller School of Medicine, Miami, Florida; 5 University of Miami Institute for Advanced Study of the Americas, Coral Gables, Florida; 6 Department of Public Health Sciences, University of Miami, Miami, Florida; 7 Department of Medicine, Division of Internal Medicine, University of Miami, Miami, Florida; 8 Department of Surgery, University of Washington, Seattle; 9 World Health Organization, Geneva, Switzerland

## Abstract

**Importance:**

Unmet social needs in local populations may hinder the development of targeted cancer control interventions aimed at improving screening utilization and early-stage breast cancer diagnosis to ultimately improve breast cancer survival disparities.

**Objective:**

To evaluate if (1) city-funded screening mammography is associated with utilization of screening mammography, (2) unmet social needs are associated with utilization of screening mammography, and (3) unmet social needs are associated with later-stage disease at diagnosis.

**Design, Setting, and Participants:**

This cohort study included patients with stages I-IV invasive ductal or lobular carcinoma treated at an academic medical center (including both an underserved safety-net hospital [SNH] and a National Cancer Institute–designated academic cancer center [ACC]) from 2020 to 2023. Eligible patients were aged 18 years or older and able to consent. Data were analyzed between July 2023 and September 2023.

**Exposure:**

The Health Leads Social Needs Screening Toolkit, a screening tool that gathers information on the most common social need domains affecting patient health.

**Main Outcomes and Measures:**

Univariable and multivariable logistic regression was utilized to evaluate the following primary outcomes: (1) routine screening mammography and (2) American Joint Committee on Cancer 8th edition clinical stage at presentation.

**Results:**

Of the 322 women who completed the Health Leads Social Needs Screening Toolkit, 201 (62%) self-identified as Hispanic, 63 (19%) as non-Hispanic Black, and 63 (19%) as non-Hispanic White. Two hundred fifty-five (76%) patients with access to city-funded screening mammography completed a screening mammogram. Patients who presented to the SNH were more likely to present with late-stage disease compared with early-stage disease (15 of 48 [31%] vs 50 of 274 [18%]; *P* = .04). On multivariable logistic regression, not completing a screening mammography was associated with having an increasing number of unmet social needs (OR, 0.74; 95% CI, 0.55-0.99; *P* = .047) and an increasing age at diagnosis (OR, 0.92; 95% CI, 0.89-0.96; *P* < .001). Moreover, increasing unmet social needs was significantly associated with late-stage diagnosis above and beyond screening mammography (OR, 1.38; 95% CI, 1.01-1.89; *P* = .04).

**Conclusions and Relevance:**

In this cohort study, access to screening mammography did not translate to utilization of screening mammography, increasing unmet social needs were significantly associated with lower rates of screening mammography, and those with increasing unmet social needs were more likely to present with late-stage disease. This association transcended recruitment site (SNH vs ACC), indicating that patients in either hospital setting may benefit from unmet social needs screening to overcome access to care barriers associated with late-stage disease at diagnosis.

## Key Points

**Question:**

Are unmet social needs associated with breast cancer screening mammography utilization and stage at diagnosis?

**Findings:**

In this cohort study of 322 patients with stage I-IV breast cancer, having an increasing number of unmet social needs was significantly associated with decreased screening mammography utilization and late-stage diagnosis.

**Meaning:**

These results suggest that unmet social needs should be screened at intake at all hospital systems to promote screening utilization and early-stage diagnosis.

## Teaser

This cohort study examines the association of patients with unmet social needs with screening mammography utilization and stage of diagnosis among patients with breast cancer.

## Introduction

Despite improvements in screening mammography, diagnosis, and treatment, breast cancer survival disparities persist among vulnerable populations, even among those living in high-income countries.^1,2^ One reason is that women living in disadvantaged neighborhoods continue to present with later-stage disease (stage III/IV) breast cancer, which compared with early-stage breast cancer (stage I/II) is ultimately associated with shorter survival.^2,3^

There is growing recognition that social determinants of health (SDOH) are some of the strongest contributors to health inequalities within the US and around the world.^1,2,3,4^ Specifically, SDOH are defined by the World Health Organization as the contextual-level conditions in which individuals are born, grow, work, live, and age and the wider sets of forces and systems shaping the conditions of life.^5^ Although SDOH are key to health outcomes, they are often the upstream mediators of social needs, which reflect direct individual-level factors associated with health outcomes.

Unmet social needs are downstream mediators of SDOH that are often more tangible and modifiable for health systems and physicians to address in their patients (Figure).^6^ Unmet social needs, such as housing instability, social isolation, food insecurity, and transportation challenges directly affect a patient’s ability to access care and have been associated with poorer health outcomes.^7,8,9^ Individuals with cancer often report at least 1 unmet social need, especially within financial, informational, psychological, and physical domains.^10^ However, there is a critical knowledge gap evaluating the impact of unmet social needs on utilization of screening mammography and breast cancer stage at diagnosis, particularly in high-income settings with access to screening mammography.^11,12,13,14,15^

**Figure.**
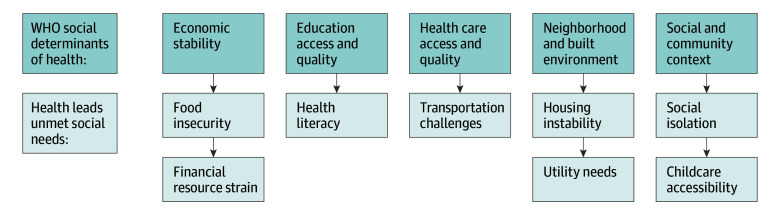
Relationship of World Health Organization (WHO) Social Determinants of Health and Unmet Social Needs

To address these gaps, the main objectives of this study were to evaluate (1) if city-funded screening mammography is associated with utilization of screening mammography, (2) if unmet social needs are associated with utilization of screening mammography, and (3) if unmet social needs are associated with later-stage disease at diagnosis above and beyond established risk-factors for late-stage disease (eg, age, race and ethnicity, insurance status, lack of screening mammography) in a high-income country where the local population is socioeconomically, racially, and ethnically diverse and has free access to screening mammography. Specifically, all women 200% below poverty level in Miami-Dade County, where this study takes place, have access to free screening mammography.

To accomplish these objectives, we evaluated unmet social needs using the validated Health Leads Social Need Screening Toolkit in patients enrolled in our Miami Breast Cancer Disparities Study, a prospective epidemiologic cohort study that enrolls newly diagnosed women with stage I-IV breast cancer treated at our National Cancer Institute-Academic Cancer Center (ACC) and adjacent sister safety-net hospital (SNH) in Miami-Dade County. We hypothesize that (1) free screening mammography availability does not ensure utilization, (2) patients with more unmet social needs will have less utilization of screening mammography, and (3) patients with more unmet social needs will have higher odds of late-stage disease at presentation compared with patients with little to no unmet social needs, even if these patients had a history of or access to screening mammography.^16^

## Methods

### Study Design and Setting

This is a prospective, survey-based cohort study of newly diagnosed patients with breast cancer treated at an ACC (University of Miami Sylvester Comprehensive Care Center, an NCI-designated Cancer Center) and its partner SNH (Jackson Health System) from 2020 to 2023. Patients without insurance who live below the National Federal Poverty Level and can prove residency in Miami-Dade County are eligible to receive care at the SNH through a Jackson Card.^17,18^

All patients aged 18 years and older with stages I to IV invasive ductal or lobular carcinoma who were able to consent were included. English and Spanish speaking patients were included. Women with ductal carcinoma in situ or other malignancies of the breast (ie, sarcoma, lymphoma) were excluded. Enrolled women completed the Health Leads Social Needs Screening Toolkit. This study followed the Strengthening the Reporting of Observational Studies in Epidemiology (STROBE) reporting guidelines. This study was approved by the University of Miami and Jackson Health System institutional review boards and complied with all relevant ethical regulations. Participants provided written informed consent.

### Primary Exposure

Unmet social needs were measured using the validated Health Leads Social Needs Screening Toolkit.^19^ The Health Leads Social Needs Screening Toolkit is a screening tool that queries the most common social need domains that affect patient health. In addition to including essential social need domains—food insecurity, housing instability, utility needs, financial resource strain, and transportation challenges—this survey was adapted to include questions on the optional social need domains of childcare accessibility, health literacy, and social isolation (Figure).^15^ In addition, patients provided sociodemographic information and breast cancer history.

### Primary Outcomes

The primary outcomes were routine screening mammography and clinical American Joint Committee on Cancer 8th edition clinical stage at presentation. Screening mammography was confirmed through patient interview and medical record review. Stage was determined based on electronic medical record review. Patients with missing stage were not included in the analysis.

### Covariates

Covariates were included based on prior research to control for factors associated with screening mammography and late-stage at diagnosis. They included granular individual-level factors (age, number of children supported under 18, household number, race and ethnicity, primary language spoken, relationship status, employment status, annual household income [quartiles], highest education, country of birth, insurance status, history of routine screening mammography, self-reported unmet social needs [as assessed by the Health Leads Social Needs Screening Toolkit]) and institutional-level factors (recruitment site). It is important to note that all women 200% below the poverty level in Miami-Dade County, where the study takes place, have free access to screening mammography.^16^ This offers an opportunity for our team to identify unmet targets associated with stage at diagnosis, beyond access to screening mammography.

Age at diagnosis was treated as a continuous variable and was included as younger women have been shown to have higher-stage at presentation.^20^ Patient self-identified race and ethnicity was categorized into non-Hispanic White, non-Hispanic Black, and Hispanic (any race). Self-identified race and ethnicity was included because prior studies have found a higher proportion of Black women live in low-income neighborhoods and have a higher incidence of more aggressive tumor subtypes (triple negative breast cancer) that may lead to later stage at presentation.^21,22,23^ Insurance was categorized into private or Medicare; Medicaid, Jackson Card, state-sponsored, or military; and uninsured, self-pay, Indian Health Service, unsure, or other. Insurance status was included as lack of insurance has been associated with living in a low-income neighborhood and studies have established that the uninsured are more likely to present with late-stage disease.^24,25,26,27,28^

### Statistical Analysis

We characterized individual-level factors (age, number of children supported under age 18 years, household number, race and ethnicity, primary language spoken, relationship status, employment status, annual household income [quartiles], highest education, country of birth, insurance status, and institutional-level factors [recruitment site]) by (1) yes or no screening mammography and (2) self-reported unmet social needs (as assessed by the Health Leads Social Needs Screening Toolkit) using χ^2^ tests and analysis of variance (ANOVA). Patients who were enrolled in the prospective study but did not complete the Health Leads Social Needs Screening Toolkit were not included in the analysis.

Cronbach α was calculated for all items of the Health Leads Social Needs Screening Toolkit to test the internal consistency of the survey items, or how closely related the set of items are as a group.^29^ Univariable and multivariable logistic regression was performed to determine factors associated with screening mammography utilization while accounting for individual-level factors (age, race, ethnicity, highest education, work situation, household number, number of children supported under age 18 years, and self-reported unmet social needs [as assessed by the Health Leads Social Needs Screening Toolkit]), and recruitment site. Separate univariable and multivariable logistic regressions were performed to determine factors associated with late-stage (American Joint Committee on Cancer 8th edition) at diagnosis early (I or II) vs late (III or IV) while accounting for individual-level factors (age, race, ethnicity, highest education, work situation, household number, number of children supported under age 18 years, screening mammography, and self-reported unmet social needs (as assessed by the Health Leads Social Needs Screening Toolkit), and recruitment site.

STATA version 17 (StataCorp LLC) was used for statistical analysis. All statistical tests were 2-sided, and statistical significance was assessed at α less than .05.

## Results

### Patient Characteristics and Screening Mammography Utilization

The study sample consisted of 505 patients, of which 336 (67%) completed the Health Leads Social Needs Screening Toolkit and were included in the analysis. The Health Leads Social Needs Screening Toolkit had good internal validity in our study population (α = 0.68). A total of 201 patients (62%) self-identified as Hispanic, 63 (19%) as Black, and 63 (19%) as White (Table 1). Overall, 255 (76%) of the population had a screening mammography.

**Table 1.** Patient Characteristics by History of Routine Screening Mammography

Characteristics	Patients, No. (%)			*P* value	*df*
	Total (n = 336)	History of screening mammography (n = 255)	No history of screening mammography (n = 81)		
Unmet social needs					
0 Unmet social needs	197 (59)	144 (57)	53 (65)	.34	2
1 Unmet social need	72 (21)	57 (22)	15 (19)		
2 Or more unmet social needs	67 (20)	54 (21)	13 (16)		
Age diagnosed, mean (SD), y	55.4 (11.4)	58.0 (9.6)	47.7 (13.2)	<.001	331
No. of children under 18 y supporting, mean (SD)	2.62 (1.6)	2.5 (1.5)	3.1 (1.9)	.06	334
No. of people living in household, mean (SD)	0.51 (0.9)	0.4 (0.7)	1.0 (1.3)	<.001	334
Recruitment site					
Safety-net hospital	69 (21)	49 (19)	20 (25)	.30	1
Academic cancer center	266 (79)	205 (81)	61 (75)		
Race and ethnicity					
Hispanic	201 (62)	156 (63)	45 (58)	.15	2
Non-Hispanic White	60 (19)	48 (20)	12 (15)		
Non-Hispanic Black	63 (19)	42 (17)	21 (27)		
Primary language spoken					
English	191 (58)	141 (56)	50 (63)	.28	1
Spanish	138 (42)	109 (44)	29 (37)		
Relationship status					
Married	151 (45)	113 (44)	38 (47)	<.001	2
Divorced, widowed, or single	107 (32)	98 (38)	9 (11)		
Never married/part of an unmarried couple	78 (23)	44 (17)	34 (42)		
Employment status					
Employed	174 (52)	128 (50)	46 (57)	.21	2
Unemployed	52 (16)	37 (15)	15 (19)		
Other^a^	109 (33)	89 (35)	20 (25)		
Annual household income quartiles					
≤$15 999	70 (21)	54 (21)	16 (20)	.63	4
$16 000-$49 000	86 (26)	60 (24)	26 (32)		
$50 000-$99 999	69 (21)	53 (21)	16 (20)		
≥$100 000	59 (17)	47 (18)	12 (15)		
Do not know or refuse to answer	52 (16)	41 (16)	11 (14)		
Education level					
None to some high school	37 (11)	27 (11)	10 (12)	.92	2
High school graduate to some college or technical school	154 (47)	116 (47)	38 (47)		
College graduate	139 (42)	106 (43)	33 (41)		
Country of birth					
Born outside US	227 (68)	180 (71)	47 (58)	.04	1
US born	109 (32)	75 (29)	34 (42)		
Insurance					
Private or Medicare	198 (59)	155 (61)	43 (53)	.26	2
Medicaid, Jackson Card, state-sponsored, military	113 (34)	84 (33)	29 (36)		
Uninsured, self-pay, Indian Health Service, unsure, or other	25 (7)	16 (6)	9 (11)		

^a^ Other includes retired, disabled, unpaid primary caregiver, and student.

### Unmet Social Needs and Screening Mammography Utilization

On multivariable regression, after accounting for age at diagnosis, race, ethnicity, employment status, education level, history of screening mammography, insurance type, number of children supporting in household under age 18 years, number of people in household, number of unmet social needs, and recruitment site, factors associated with not having a screening mammography were having an increasing number of unmet social needs (OR, 0.74; 95% CI, 0.55-0.99; *P* = .047) and increasing age at diagnosis (OR, 0.92; 95% CI, 0.89-0.96; *P* < .001) (Table 2). There was no significant association between a specific unmet social need and use of screening mammography (eFigure 1 in Supplement 1).

**Table 2.** Univariable and Multivariable Regression for Variables Associated With Not Having Screening Mammography Utilization

Variables	Univariable		Multivariable	
	OR (95% CI)	*P* value	OR (95% CI)	*P* value
No. of unmet social needs^a^	0.91 (0.73-1.13)	.39	0.74 (0.55-0.99)	.047
No. of children under 18 supporting^a^	2.06 (1.60-2.67)	<.001	1.31 (0.84-2.05)	.23
No. of people in household^a^	1.29 (1.12-1.47)	<.001	1.03 (0.8-1.31)	.84
Age diagnosed^a^	0.93 (0.91-0.95)	<.001	0.92 (0.89-0.96)	<.001
Recruitment site				
Academic cancer center	1 [Reference]	[Reference]	1 [Reference]	[Reference]
Safety-net hospital	1.65 (1.10-2.48)	.015	1.20 (0.53-2.71)	.67
Race				
White	1 [Reference]	[Reference]	1 [Reference]	[Reference]
Black	0.88 (0.55-1.42)	.61	0.57 (0.21-1.58)	.28
Ethnicity				
Non-Hispanic	1 [Reference]	[Reference]	1 [Reference]	[Reference]
Hispanic	1.07 (0.72-1.61)	.74	1.22 (0.54-2.79)	.63
Employment status				
Employed	1 [Reference]	[Reference]	1 [Reference]	[Reference]
Not working	0.76 (0.49-1.19)	.24	1.34 (0.64-2.81)	.44
Education				
Some college or college graduate	1 [Reference]	[Reference]	1 [Reference]	[Reference]
Some high school or high school graduate	1.15 (0.71-1.88)	.57	1.16 (0.53-2.54)	.71
Insurance				
Private insurance	1 [Reference]	[Reference]	1 [Reference]	[Reference]
Medicare or military	0.55 (0.31-0.98)	.044	0.85 (0.37-1.93)	.69
Medicaid or Jackson Card	1.38 (0.77-2.48)	.28	1.08 (0.41-2.87)	.87

^a^ Continuous variable.

### Patient Characteristics and Unmet Social Needs

Patients who identified 2 or more unmet social needs were more likely to present with late-stage disease compared with early-stage disease (16 of 48 [33%] vs 48 of 274 [18%]; *P* = .04) (Table 3). Specifically, patients having unmet social needs in the domains of utility needs or childcare accessibility were more likely to present with late-stage disease compared with early-stage disease (utilities, 8 of 48 [17%] vs 14 of 273 [5%]; *P* = .004; childcare, 5 of 43 [12%] vs 8 of 244 [3%]; *P* = .02) (eFigure 2 in Supplement 1). Patients with late-stage diagnosis supported more children under the age of 18 years compared with patients with early-stage diagnosis (mean [SD], 3.08 [2.1] vs 2.6 [1.5]; *P* = .008). Patients who presented to the SNH were more likely to present with late-stage disease compared with early-stage disease (15 of 48 [31%] vs 50 of 274 [18%]; *P* = .04). Patients who ever had a previous mammography screening were more likely to present with early-stage disease vs late-stage disease (213 of 274 [78%] vs 30 of 48 [63%]; *P* = .02).

**Table 3.** Patient Characteristics by Breast Cancer Stage at Presentation

Characteristic	Patients, No. (%)			*P* value	*df*
	Total (n = 322)	Early-stage (n = 274)	Late-stage (n = 48)		
Unmet social needs					
0	189 (59)	166 (61)	23 (48)	.04	2
1	69 (21)	60 (22)	9 (19)		
≥2	64 (20)	48 (18)	16 (33)		
Age diagnosed, mean (SD), y	55.4 (11.4)	55.7 (11.1)	53.4 (12.7)	.15	317
No. of children under 18 supporting, mean (SD)	2.62 (1.6)	2.60 (1.5)	3.08 (2.1)	.008	320
No. of people living in household, mean (SD)	0.51 (0.9)	0.60 (1.0)	0.49 (0.8)	.32	320
Recruitment site					
Safety-net hospital	65 (20)	50 (18)	15 (31)	.04	1
Academic cancer center	256 (80)	223 (82)	33 (69)		
Race and ethnicity					
Hispanic	193 (62)	168 (63)	25 (56)	.27	2
Non-Hispanic White	55 (18)	48 (18)	7 (16)		
Non-Hispanic Black	62 (20)	49 (19)	13 (29)		
Primary language spoken					
English	183 (58)	155 (58)	28 (60)	.82	1
Spanish	132 (42)	113 (42)	19 (40)		
Has patient ever had a screening mammography					
Yes	243 (76)	213 (78)	30 (63)	.02	1
No	78 (24)	60 (22)	18 (38)		
Relationship status					
Married	146 (45)	121 (44)	25 (52)	.59	2
Divorced, widowed, or single	192 (32)	89 (33)	13 (27)		
Never married/part of an unmarried couple	74 (23)	64 (23)	10 (21)		
Employment status					
Employed	164 (51)	143 (52)	21 (45)	.51	2
Unemployed	52 (16)	42 (15)	10 (21)		
Other^a^	105 (33)	89 (33)	16 (34)		
Annual household income quartiles					
≤$15 999	68 (21)	55 (20)	13 (27)	.57	4
$16 000-$49 000	84 (26)	70 (26)	14 (29)		
$50 000-$99 999	69 (21)	62 (23)	7 (15)		
≥$100 000	52 (16)	46 (17)	6 (13)		
Do not know/refused to answer	49 (15)	41(15)	8 (17)		
Education level					
None to some high school	36 (11)	31 (12)	5 (11)	.44	2
High school graduate to some college or technical school	148 (47)	122 (45)	26 (55)		
College graduate	132 (42)	116 (43)	16 (34)		
Country of birth					
Born outside US	218 (68)	188 (69)	30 (63)	.40	1
US born	104 (32)	86 (31)	18 (38)		
Insurance					
Private or Medicare	188 (58)	164 (60)	24 (50)	.06	2
Medicaid, Jackson Card, state-sponsored, or military	111 (35)	88 (32)	23 (48)		
Uninsured, self-pay, Indian Health Service, unsure, or other	23 (7)	22 (8)	1 (2)		

^a^ Other included individuals who were retired, disabled, unpaid primary caregivers, or students.

### Unmet Social Needs and Stage at Presentation

On multivariable regression, after accounting for age at diagnosis, race, ethnicity, employment status, education level, history of screening mammography, insurance type, number of children supporting in household under age 18 years, number of people in household, number of unmet social needs, and recruitment site, factors associated with late-stage at diagnosis were having an increasing number of unmet social needs (OR, 1.38; 95% CI, 1.01-1.89; *P* = .04) (Table 4).

**Table 4.** Univariable and Multivariable Regression for Variables Associated With Late-Stage Breast Cancer at Presentation

Variables	Univariable		Multivariable	
	OR (95% CI)	*P* value	OR (95% CI)	*P* value
No. of unmet social needs^a^	1.32 (1.04-1.66)	.02	1.38 (1.01-1.89)	.04
No. of children under 18 supporting^a^	1.43 (1.09-1.88)	.01	1.25 (0.75-2.06)	.39
No. of people in household^a^	1.20 (1.02-1.42)	.03	1.09 (0.82-1.44)	.56
Age diagnosed^a^	0.98 (0.96-1.01)	.21	1.03 (0.98-1.08)	.30
Recruitment site				
Academic cancer center	1 [Reference]	[Reference]	1 [Reference]	[Reference]
Safety-net hospital	2.04 (1.07-3.89)	.03	1.53 (0.58-4.04)	.39
Race				
White	1 [Reference]	[Reference]	1 [Reference]	[Reference]
Black	2.19 (1.11-4.34)	.03	1.02 (0.26-4.03)	.10
Ethnicity				
Non-Hispanic	1 [Reference]	[Reference]	1 [Reference]	[Reference]
Hispanic	0.66 (0.37-1.18)	.16	0.82 (0.24-2.82)	.75
Employment status				
Employed	1 [Reference]	[Reference]	1 [Reference]	[Reference]
Not working	1.42 (0.95-2.04)	.23	2.39 (0.86-6.58)	.09
Education				
Some college or college graduate	1 [Reference]	[Reference]	1 [Reference]	[Reference]
Some high school or high school graduate	1.63 (0.90-2.94)	.11	1.09 (0.43-2.78)	.85
Previous mammography screening				
Yes	1 [Reference]	[Reference]	1 [Reference]	[Reference]
No	2.40 (1.31-4.39)	.005	2.28 (0.83-6.27)	.11
Insurance				
Private insurance	1 [Reference]	[Reference]	1 [Reference]	[Reference]
Medicare or military	0.88 (0.35-2.22)	.79	1.17 (0.31-4.39)	.82
Medicaid or Jackson Card	2.80 (1.35-5.82)	.006	1.31 (0.44-3.85)	.63

^a^ Continuous variable.

## Discussion

This prospective cohort analysis takes a 3-pronged approach to understand how unmet social needs affect screening mammography utilization and breast cancer stage at diagnosis in a high-income country hospital setting that serves a socioeconomically and racial and ethnically diverse population, with city-funded access to screening mammography. We found that having access to screening mammography did not ensure utilization, that increasing unmet social needs were associated with less screening mammography utilization, and that increasing unmet social needs were associated with late-stage diagnosis, even after controlling for covariates such as screening mammography.

As we saw in this population, although access to screening mammography is accessible for patients in our population, it did not necessarily translate to significant utilization of screening mammography across the entire population. In our study, patients with more household members and unmarried patients were less likely to obtain screening mammography. An in-depth review of barriers to screening mammography by race and ethnicity by Miller et al^30^ categorized barriers into types: psychological or knowledge-related barriers, logistical barriers, and culture or immigration-related barriers. The factors identified in our study fall under psychological or knowledge-related barriers, specifically prioritization. Competing priorities such as caregiving, especially with less social support such as a spouse, may prevent utilization of screening mammography even if it is accessible. Our finding that insurance access was not significantly associated with screening mammography further highlights this point. When considering the racial and ethnic diversity of our cohort, we found that although our rates for White and Black patients are close to nationally reported rates, our screening mammography rates among Hispanics was higher than that seen in other studies.^31^ These findings may reflect our location in a majority-minority population geared toward Hispanics where access to care and utilization may be higher due to a large Spanish-speaking population among clinicians at our institutions.^32,33^ Along the same lines, those who were born outside the US were more likely to undergo screening mammography. While citizenship and primary language other than English from patients born in another country have been cited as barriers to screening mammography in Hispanic populations, our unique location in South Florida, which consists of a higher percentage of foreign-born residents, is an interesting contrast to previous studies.

To further understand why availability of screening mammography may not translate to utilization, we evaluated the association between unmet social needs and screening mammography. We identified that an increasing amount of unmet social needs had decreased odds of receiving a screening mammography, even after controlling for individual-level confounders and access to care barriers such as insurance status. The reasons behind this finding are multifactorial. Increased unmet social needs may overwhelm patients so that they are unable to find the time or resources to schedule preventative health check-ups.^34,35^ Additionally, negative attitudes toward prevention care or medical mistrust may also influence screening mammography utilization.^36^ Interestingly education level did not impact screening utilization, suggesting other influences such as personal beliefs, fear, and medical mistrust may play a more important role than lack of knowledge. Future analyses should evaluate which group of unmet social needs most correlates with not obtaining a mammogram to inform interventions.

Finally, our third objective was to evaluate whether having a greater burden of unmet social needs was associated with late-stage diagnosis. We found that the only factor significantly associated with late-stage disease was increasing number of unmet social needs beyond (and after controlling for) age at diagnosis, race, ethnicity, employment, education, history of screening mammography, insurance, number of children supporting under age 18 years, number of people in household, and recruitment site. Moreover, although lack of routine screening mammography was independently associated with later-stage disease at presentation on univariable analysis, it was not significant on multivariable analysis. This suggests that factors underlying utilization of screening mammography are being accounted for by unmet social needs.

Specifically, the Health Leads Toolkit assesses different aspects of patients’ lives such as housing instability and food insecurity, childcare accessibility, financial resource strains (such as medical costs), health literacy, transportation challenges, and social isolation, all of which may interfere with use of preventative medical services. Therefore, implementation of a social needs screening by social workers or community health workers may help identify specific barriers for patients that are addressable to not only identify but also increase preventative health behaviors and earlier detection in vulnerable populations that are at risk of having unmet social needs. Adoption of global strategies such as pillar 1 of the Global Breast Cancer Initiative (“Health promotion for early diagnosis”) can guide future studies that evaluate methods to overcome this utilization barrier and increase the proportion of early vs late-stage breast cancers (stage-shifting).^37^ For example, health promotion through increasing awareness supporting routine screening mammography and implementation of easily adoptable adjunct mechanisms for earlier detection, such as clinical breast examinations, are 2 mechanisms that can lead to stage-shifting. In a high-income country, the Canadian National Breast Cancer Screening Studies 25-year update found no survival benefit for women undergoing screening mammography; however, this might be attributed to the successful early detection strategies employed for the control group, namely breast health education and clinical breast examination.^38,39,40^ Moreover, studies in low- and middle-income countries, namely the Trivandrum^41^ and Mumbai^42^ clinical breast examination screening trials, found that awareness and education of clinical breast examinations promoted earlier detection of disease, shifting the stage of detection of tumors to a lower stage. It is important to note that the Trivandrum and Mumbai studies had different population characteristics, screening protocols, and follow-up intervals, and only the Mumbai study found significant mortality reduction in women over 50 years old. These studies did not suggest forgoing screening mammography, but rather that in a reasonable and appropriate population, implementing clinical breast examinations can contribute to stage-shifting and improve early-stage breast cancer detection. This might be an approach to consider in certain vulnerable populations in high-income countries, like those with a high burden of unmet social needs.

While we present practical options to implement in clinical practices or communities, the onus of reducing health disparities by addressing unmet social needs cannot fall solely on health care professionals and systems. Health care policies addressing increased access to screening mammography for groups with the lowest rates of screening, namely patients in rural areas and historically marginalized racial and ethnic groups.^43^ Accessibility on a health policy level increasingly requires changes such as lowering the cost of screening, expanding insurance coverage (as screening rates for uninsured women are far lower than insured), and increasing funding to community health centers that serve low-income patients.^43^ Once patients get diagnosed, the cost and coverage for additional procedures such as imaging, biopsy, and surgery are all additional barriers that must be addressed. National organizations such as the American Cancer Society have posited multi-level frameworks for policy changes to address cancer disparities that assess both top-down and bottom-up social determinants of health.^44^

### Limitations

Overall, our study has inherent limitations and strengths. The study cohort was recruited from patients presenting to 2 hospitals on the same medical campus in South Florida, limiting generalizability. However, the ACC and SNH are distinct in the populations they capture and serve very different socioeconomic, insurance, and geographic populations of Miami-Dade County.^45^ In addition, comparison of our patient population with the Florida Cancer Database System, which includes all breast cancer cases from Miami-Dade County, shows that our data reflect county-level data with respect to race, ethnicity, and clinical stage when compared with the Florida Cancer Data System breast cancer county-level data, suggesting a representative population-based sample. Our study initially took place over a significant historical period in health care, the COVID-19 pandemic, which affected health behaviors and access to preventative screening for many patients, especially Black and Latinx women in the US.^46^

Although this study was prospective, it is still susceptible to response and recall bias. Additionally, because our study did not include or collect data on patients with ductal carcinoma in situ, this may have biased our analysis of screening mammography penetrance in the population. Despite these limitations, our study is unique in its ability to capture and analyze the impact of granular individual-level social and survey data not available in national databases such as number of children supported under age 18 years, household number, primary language spoken, relationship status, employment status, annual household income (quartiles), highest education, country of birth, insurance status, history of routine screening mammography, and self-reported unmet social needs as assessed by the Health Leads Social Needs Screening Toolkit and institutional-level factors (recruitment site) on stage at diagnosis above and beyond access to screening mammography.

## Conclusions

This prospective cohort study evaluated the association of unmet social needs with screening mammography utilization and breast cancer stage at presentation. We found that access to screening mammography does not ensure utilization and that an increased number of unmet social needs was associated with decreased screening mammography. Moreover, increasing unmet social needs was significantly associated with late-stage diagnosis, above and beyond historically recognized factors such as access to insurance and screening mammography. This novel research is important as diagnosis at a later stage significantly affects survival outcomes, and interventions that promote stage-shifting are critical. By incorporating social needs screening into health care and developing interventions to overcome them, breast cancer outcomes can be improved. This study therefore reveals a need for integration of social needs screening measures in the health care setting to identify unmet social needs. In turn, partnerships between hospitals, social workers, and communities should aim to develop targeted interventions to overcome barriers to screening utilization and unmet social needs to increase early-stage diagnosis, a direct mediator of improved survival outcomes, even in high-income countries with access to screening mammography.

## Notes

Supplement 1. **eFigure 1.** Specific Unmet Social Needs by History of Screening Mammography **eFigure 2.** Specific Unmet Social Needs by Stage at Presentation

Supplement 2.
Data Sharing Statement
