# Supplementary material for: Unmet Social Needs and Breast Cancer Screening Utilization and Stage at Presentation
Source: JAMA Netw Open. 2024 Feb 14;7(2):e2355301. doi: 10.1001/jamanetworkopen.2023.55301 (PMC10867685; doi:10.1001/jamanetworkopen.2023.55301)
Supplement: Supplement 1. — eFigure 1. Specific Unmet Social Needs by History of Screening Mammography eFigure 2. Specific Unmet Social Needs by Stage at Presentation [file jamanetwopen-e2355301-s001.pdf]

Supplemental Online Content

Goel N, Lubarsky M, Hernandez AE, et al. Unmet social needs and breast cancer screening utilization and stage at presentation. *JAMA Netw Open.* 2024;7(2):e2355301. doi:10.1001/jamanetworkopen.2023.55301

eFigure 1. Specific Unmet Social Needs by History of Screening Mammography

eFigure 2. Specific Unmet Social Needs by Stage at Presentation

This supplemental material has been provided by the authors to give readers additional information about their work.

© 2024 Goel N et al. *JAMA Network Open.*

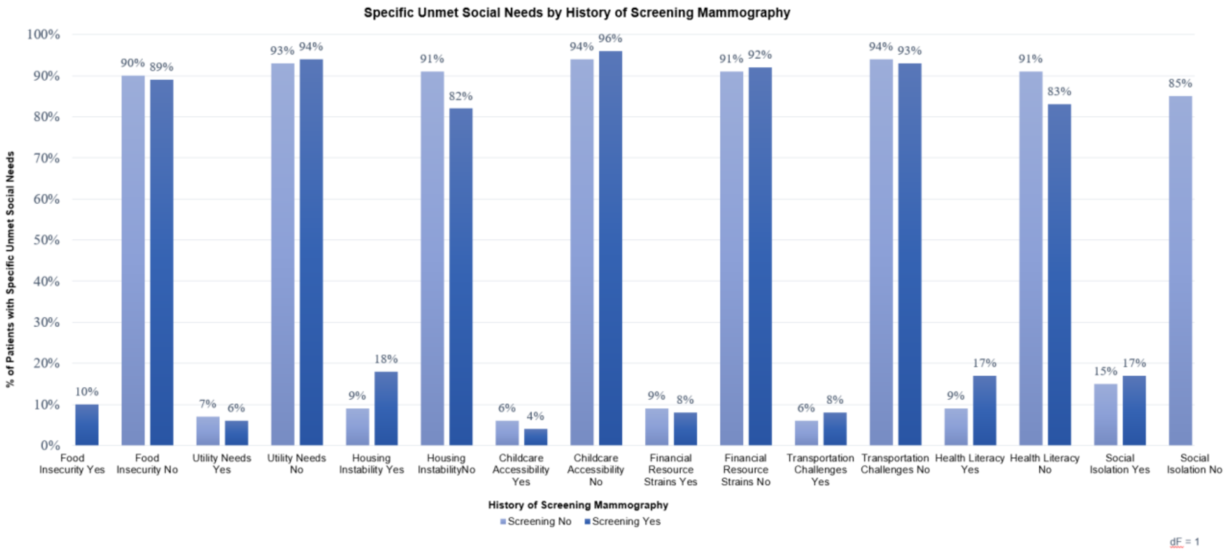

eFigure 1. Specific Unmet Social Needs by History of Screening Mammography

© 2024 Goel N et al. *JAMA Network Open.*

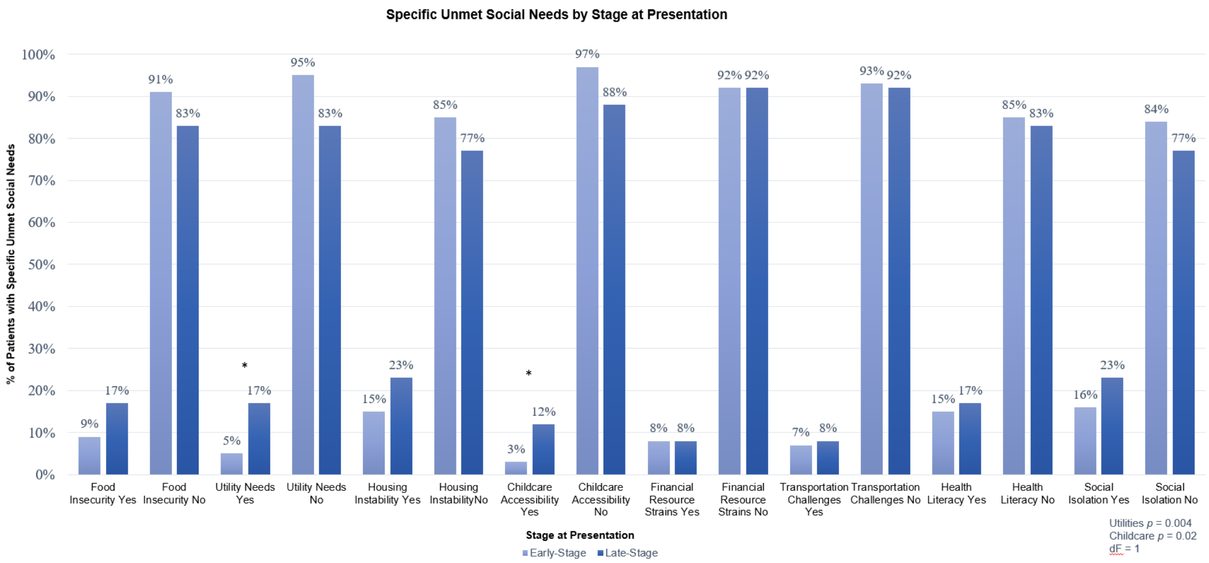

eFigure 2. Specific Unmet Social Needs by Stage at Presentation

© 2024 Goel N et al. *JAMA Network Open.*
