# Supplementary material for: Unmet Social Needs and Breast Cancer Screening Utilization and Stage at Presentation
Source: JAMA Netw Open. 2024 Feb 14;7(2):e2355301. doi: 10.1001/jamanetworkopen.2023.55301 (PMC10867685; doi:10.1001/jamanetworkopen.2023.55301)
Supplement: Supplement 2. — Data Sharing Statement [file jamanetwopen-e2355301-s002.pdf]

Data Sharing Statement

Goel. Unmet Social Needs and Breast Cancer Screening Utilization and Stage at Presentation. *JAMA Netw Open*. Published February 08, 2024. doi:10.1001/jamanetworkopen.2023.55301

Data
Data available: No

Additional Information
Explanation for why data not available: Upon request to the PI.
